# Autoimmune hepatitis and juvenile systemic lupus erythematosus: 2 for 1?

**DOI:** 10.1186/1546-0096-12-S1-P336

**Published:** 2014-09-17

**Authors:** Mariana Rodrigues, Francisca Aguiar, Eunice Trindade, Marta Tavares, Jorge Amil, Iva Brito

**Affiliations:** 1 Pediatric Rheumatology, Centro Hospitalar São João, Porto, Portugal; 2 Rheumatology, Centro Hospitalar São João, Porto, Portugal; 3 Pediatric Gastroenterology, Centro Hospitalar São João, Porto, Portugal

## Introduction

Autoimmune hepatits (AIH) is characterized by increased liver enzymes, hypergammaglobulinemia, specific autoantibodies and typical hystologic findings. Extrahepatic autoimmune phenomena may coexist with type 1 AIH.

Systemic lupus erythematosus (SLE) is a chronic multisystem autoimmune disease associated with the production of autoantibodies. Although it has the potential to affect any organ, clinically significant hepatic involvement is considered to be uncommon and liver dysfunction is not a diagnostic criteria.

## Objectives

The authors present a clinical case and discuss the challenges posed by the differential diagnosis between AIH type 1 and SLE.

## Methods

A previously healthy 12 year-old girl was referred to our Gastroenterology Unit due to the incidental finding of high transaminases (30 fold upper limit of normal (ULN)). Besides fatigue there were no other symptoms, and physical examination was unremarkable. Laboratory investigations showed hypergammaglobulinemia, no colestasis and negative viral serologies. Alfa-1 anti-trypsin deficiency, hemochromatosis and Wilson´s disease were excluded.

Transcutaneous biopsy revealed liver cirrhosis with chronic interface hepatitis and lymphoplasmocytic infiltrates.

AIH antibody panel was negative, including smooth muscle, liver/kidney microsome type 1, liver-specific cytosol antigen type 1 and soluble liver antigen/liver pancreas antibodies. A very high titer of antinuclear antibodies (ANA) (>1/1000) raised suspicion and led to additional studies which showed very high dsDNA antibodies and positive anti-nucleosome antibodies. The remainder antibody panel was negative, including antiphospholipid and antiribosomal P protein antibodies, with normal complement. There was a positive direct Coombs test (4 out of 5, IgG specific), without anemia or active hemolysis, normal platelets and discrete intermittent lymphopenia.

## Results

3 months after the initial presentation, malar rash and frequent oral ulcers appeared, with no renal abnormalities, photosensitivity or joint complaints.

A juvenile SLE diagnosis was thus established: lymphopenia + malar rash + oral ulcers + immunologic criteria.

After 4 months of treatment with prednisolone, azathioprin (2 mg/kg/day), hidroxychloroquin and ursodeoxycholic acid, there is a partial response with liver enzymes decreasing to 2-3 fold ULN.

## Conclusion

The distinction between SLE and type 1 AIH can be difficult, and we cannot exclude an overlap phenotype.

In the present case, the absence of liver specific antibodies and a very high titer of ANA raised the diagnostic suspicion. Cooperation with the Rheumatology Unit led to a diagnosis of SLE with implications in the monitoring, management and prognosis of the patient.

Recent case series report a much higher prevalence of liver involvement in juvenile SLE patients compared with adults, which may often precede the other symptoms.

On the other hand, juvenile patients with AIH have a significant risk of developing SLE, which calls for an increased awareness in the follow-up of these patients.

## Disclosure of interest

None declared

